# Diagnosis of infections in newborns using a new particle-mediated immunoassay for serum C-reactive protein

**DOI:** 10.1155/S146392469800025X

**Published:** 1998

**Authors:** S. Kitahashi, N. Tatsumi, S. Tagawa, T. Matsui, M. Higashihata, H. Shintaku, S. Tomoda, I. Tsuda

**Affiliations:** 1 Department of Clinical and Laboratory Medicine Osaka City University Medical School 1-5-7 Asahimachi Abeno Osaka 545 Japan; 2 Department of Clinical Hematology Osaka City University Medical School 1-5-7 Asahimachi Abeno Osaka 545 Japan; 3 Department of Pediatrics Osaka City University Medical School 1-5-7 Asahimachi Abeno Osaka 545 Japan; 4 Department of Obstetrics and Gynecology Osaka City University Medical School 1-5-7 Asahimachi Abeno Osaka 545 Japan

## Abstract

C-reactive protein (CRP) levels were measured using a new particle-mediated immunoassay. Tests for precision and linearity of this method gave satisfactory results. The minimum sensitivity of the assay was 1 ng/ml. Interference by bilirubin (<220mg/l) and haemoglobin (<20g/l) was not observed. Using this method, CRP was assayed as a means of monitoring for infection in newborns up to 72 h after delivery. The pattern of time course elevation curves was similar for both groups (10 healthy subjects and 26 patients), but the serum CRP (ng/ml) of infected newborns rose significantly higher than in healthy subjects at 24 h after birth. The rate of increase of CRP (∆CRP; ng/ml/h) may be a more useful parameter to detect infection, since a significant change in ∆CRP was apparent only 12 h after birth. The reported method was reliable and the parameters obtained were considered clinically useful for early detection of infection.


Journal of Automatic Chemistry, Vol. 20, No. 6 (November–December 1998) pp. 195–198

### Diagnosis of infections in newborns using a new particle-mediated immunoassay for serum C-reactive protein

**S. Kitahashi[1], N. Tatsumi[1,2], S. Tagawa[2], T. Matsui[1], M. Higashihata[1], H. Shintaku[3], S. Tomoda[4] and I. Tsuda[1]**

[1] *Department of Clinical and Laboratory Medicine*, [2] *Department of Clinical Hematology*, [3] *Department of Pediatrics, and* [4] *Department of Obstetrics and Gynecology, Osaka City University Medical School, 1-5-7 Asahimachi, Abeno, Osaka, 545 Japan*

*C-reactive protein (CRP) levels were measured using a new particle-mediated immunoassay. Tests for precision and linearity of this method gave satisfactory results. The minimum sensitivity of the assay was 1 ng/ml. Interference by bilirubin (<220 mg/l) and haemoglobin (<20 g/l) was not observed. Using this method, CRP was assayed as a means of monitoring for infection in newborns up to 72 h after delivery. The pattern of time course elevation curves was similar for both groups (10 healthy subjects and 26 patients), but the serum CRP (ng/ml) of infected newborns rose significantly higher than in healthy subjects at 24 h after birth. The rate of increase of CRP (ΔCRP; ng/ml/h) may be a more useful parameter to detect infection, since a significant change in ΔCRP was apparent only 12 h after birth. The reported method was reliable and the parameters obtained were considered clinically useful for early detection of infection.*

### Introduction

Three clinical parameters have been used routinely for the diagnosis of infections in infants: the number of circulating neutrophils [1]; the serum concentration of cytokines [2–4]; and the serum concentration of acute phase proteins, e.g. C-reactive protein (CRP) [5]. Among these, the measurement of CRP is particularly helpful in diagnosing early bacterial infections [6–12]. However, such measurement in newborns is not as easy as in infants, due to the difficulty of blood collection and frequent elevations of CRP. Another reason that CRP is not always measured in newborns is that the present methods for CRP determination cannot detect subtle changes.

A recently developed particle-mediated immunoassay method [13] can detect various antigens rapidly and precisely using a specific antibody reagent (Ranream). Using this method, we evaluated CRP changes for the early diagnosis of infections in newborns.

### Subjects and methods

### *Assay method*

Serum CRP was measured using an automated immunoassay system, the PAMIA-30 (Toa Medical Electronics, Kobe, Japan) [13] and a latex particle reagent coated with anti-CRP antibody (Ranream-CRP, Toa Medical Electronics). Most blood cell counters employ an impedance method to measure the number and size of blood cells, and this principle has been applied to the instrument. Principally, the size and number of aggregates resulting from a complex between antigen and antibody-coated particles are measured through the Coulter aperture. The reagent kit consists of three solutions: a buffer solution (maleic acid buffer containing 5% bovine serum albumin, 50 mM, pH 6.0), a latex reagent suspension specific for CRP, and a diluent (Tris buffer containing 3% gelatin, pH 7.5) that can be used when the CRP of the sample is very high. A commercially available WHO biologic reference sample (CRP No. 85/506) was used as our standard.

Eighty microlitres of the buffer solution was added to 10 μl of serum or plasma, and the mixture was left for 1 min. Then, 10 μl of the latex reagent was added to the above mixture. Fifteen minutes later, the agglutination that resulted from the antigen–antibody reaction was measured by PAMIA-30. When the CRP of the sample was 1000 ng/ml or higher, the sample was diluted automatically with the diluent in the reagent kit.

### *Intra- and inter-assay precision*

In order to evaluate intra-assay precision, sera with different CRP concentrations were assayed 10 times, and a coefficient variation (CV) was calculated for each serum sample. The same sera were assayed every day for two weeks, and CV was calculated to evaluate inter-assay precision.

### *Sensitivity of the CRP assay*

A standard solution of CRP (CRP 10 ng/ml, Toa Medical Electronics) was diluted with Tris buffer (pH 7.5) containing 3% gelatin to make mixtures with CRP concentrations of 0.0, 1.0, 2.0, 4.0, 6.0, 8.0 and 10.0 ng/ml. Each mixture was assayed 10 times and {(amount of polymerized particles)/(total amount of anti-CRP coated particles)} was calculated (P/T). The mean and standard deviation (SD) was calculated for each mixture.

### *Linearity*

Serum from two patients with high CRP levels (1097 ng/ml and 192 000 ng/ml) was diluted with Tris buffer (pH 7.5) containing 3% gelatin. The mean value was calculated and plotted on a graph.

0142-0453/98 $12·00 © 1998 Taylor & Francis Ltd

*Table 1. Intra- and inter-assay precision of CRP determination by counting immunoassay.*

| | Intra-assay ($n = 10$) | | | Interassay ($n = 14$) | | |
|---|---|---|---|---|---|---|
| *Sample* | *Mean (ng/ml)* | *SD(ng/ml)* | *CV (%)* | *Mean (ng/ml)* | *SD(ng/ml)* | *CV (%)* |
| 1 | 55.7 | 1.2 | 2.1 | 56.2 | 2.0 | 3.6 |
| 2 | 275.7 | 4.0 | 1.5 | 279.1 | 7.7 | 2.8 |
| 3 | 863.8 | 31.4 | 3.6 | 890.8 | 32.9 | 3.7 |

*Effect of bilirubin and haemoglobin on the CRP assay*

Bilirubin (28–220 mg/l, Interference Check A Plus Bilirubin, International Reagents, Kobe, Japan) of haemoglobin (20–50 g/l, Interference Check A Plus Haemoglobin, International Reagents) was added to serum at varying concentrations ($n = 3$), and the CRP value of each solution was compared with values obtained from the serum without added bilirubin or haemoglobin.

*Comparison of serum and plasma*

Plasma obtained from heparinized blood and serum from the same adults ($n = 10$) were assayed and the results compared.

*Correlation with results from an automated nephelometric analyser*

Serum CRP was assayed with PAMIA-30 and an automated nephelometry analyser LX3000 (Eiken Chemical, Tokyo, Japan) for randomly selected samples ($n = 50$).

*Subjects*

All newborns studied were delivered at term. A diagnosis of infection was made by clinical findings associated with fever of greater than 38 °C after delivery, as well as tachypnea, apnea, nasal flaring, retractions, cyanosis, poor skin colour, bradycardia or tachycadia. Cloudy amniotic fluid or premature rupture of membranes was also considered to be consistent with infection. Treatment with antibiotics was initiated for all infected newborns. Newborns without any signs of infection at birth were used as the non-infected control group.

The experiments were conducted with permission from our Institutional Review Board. Attending physicians obtained informed consent from the newborns' parents.

*CRP assay for non-infected and infected newborns*

CRP levels of non-infected ($n = 10$) and infected ($n = 26$) subjects were studied. Heparinized cord blood was obtained immediately after birth, and plasma was separated by centrifugation. Venous blood specimens were collected from newborns at 6, 12, 24, 48 and 72 h after birth, and serum was obtained from clotted blood within 6 h after blood collection.

*Statistics*

Student's $t$-test was used for analysis.

### Results

*Performance of CRP determination*

The intra-assay precision was 1.5–3.6% of CV, and inter-assay precision was 2.8–3.7% (table 1).

Mixtures with different CRP concentrations (0.0–10.0 ng/ml) were assayed, and mean and standard deviations of P/T were obtained. The mean +3 SD of P/T of the mixture with 0.0 ng/ml of CRP was 0.368, and this was lower than the mean −3 SD (0.369) of the mixture with 1 ng/ml of CRP. Thus, the sensitivity for CRP determination was judged to be 1 ng/ml (figure 1).

Excellent linearity was demonstrated for both samples (figure 2). Interference tests revealed bilirubin (<220 mg/l) and haemoglobin (<20 g/l) did not interfere with the measurement. CRP of serum (1206 ± 1579) and plasma (1163 ± 1597) from the same adults were not significantly different ($r^2 = 0.994$, $y = 1.008x - 52.69$, $n = 10$). The serum CRP measured by the present method correlated well with those assayed by the automated nephelometry analyser LX3000 (figure 3).

*CRP levels in non-infected and infected newborns*

No difference was observed in CRP levels between cord blood and venous blood taken immediately after birth in both infected and non-infected groups. Most individual CRP levels were less than 100 ng/ml in both. The time course of CRP in non-infected (healthy) newborns

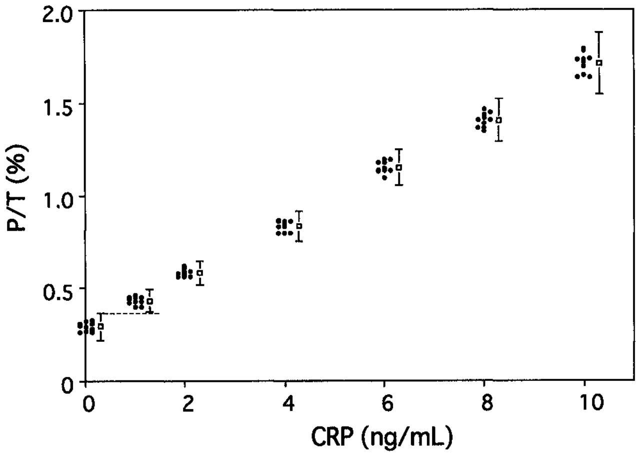

*Figure 1. Sensitivity of the CRP assay, x-axis shows concentrations of CRP and the y-axis is P/T, calculated as {(amount of polymerized particles)/(total amount of anti-CRP coated particles)}. The open circle and bars for each concentration are the means ± 3 standard deviation.*

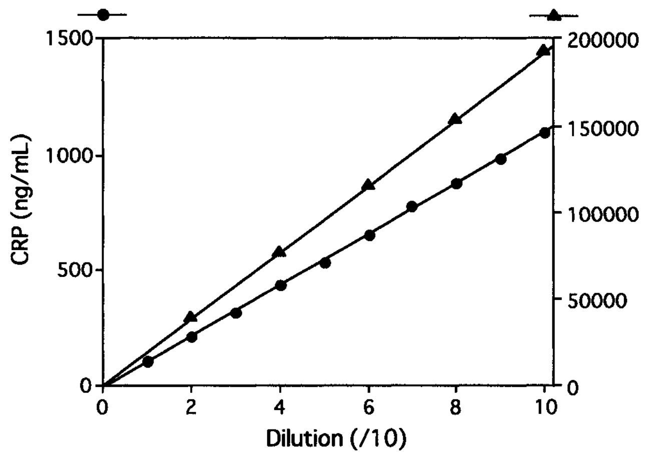

*Figure 2. Linearity; two serum samples were diluted and the CRP levels were plotted.*

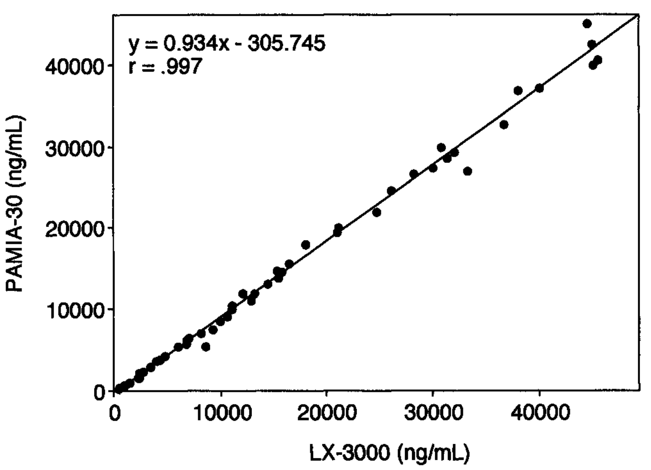

*Figure 3. Correlation with an automated nephelometry analyser LX3000.*

showed an increasing trend, reaching a maximum level by 24 h after birth, and decreasing at 48 h (table 2). Infected newborns also had increasing levels. No significant differences were seen between non-infected and infected groups up to 12 h after delivery. At 24, 48 and 72 h, CRP was significantly higher in infected newborns than in non-infected newborns.

When the ΔCRP (ng/ml/h) was calculated for both groups, a significant difference was observed between the two at 12 h.

### Discussion

The clinical utility of CRP as a marker of inflammation has increased due to precise and accurate measurements now possible with sophisticated technology, e.g. the latex particle immunoassay (LPIA) [8–10], the turbidimetric immunoassay (TIA) [5] and the radioimmunoassay (RIA) [11, 12]. These methods can determine precise levels of CRP between 50 and 5000 ng/ml, but cannot detect minor changes at lower concentrations. As a means of improving the early diagnosis and treatment of acute infection, PAMIA-30 was developed for routine clinical use.

When compared to other methods, PAMIA-30 has several advantages. The volume necessary for an assay is 40 μl, including the dead volume of 30 μl. Because it is often difficult to get enough blood for CRP assays, the advantage of PAMIA here is clearly substantial. The results can also be obtained in 15 min. Linear determination up to 200 000 ng/ml of CRP was possible. When the CRP of the samples is 1000 ng/ml or more, the PAMIA can select the dilution ratio, dilute the sample, and assay it again automatically. Both in infected and non-infected groups, the CRP level in cord blood was very low, with the CRP of two-thirds of the samples lower than 50 ng/ml in this study. With this method, such low concentrations of CRP and their changes could be detected. The results of precision tests also were satisfactory.

White blood cell counts represent an easy way to detect infection, but the white cell count usually increases after the CRP increases. Cytokines, e.g. IL-6, can also serve as a marker of infection, but they are usually limited to experimental use due to high reagent costs. Buck *et al.* [2] have reported that the measurement of IL-6 is more useful for the diagnosis of infection in infants than the measurement of CRP, and that the measurement of IL-6 levels at 24 h after birth contribute to the clinical diagnosis of infection. However, this measurement is not used very widely; measuring CRP is more practical, convenient and economical. Our data demonstrate that

*Table 2. Time course of serum CRP and ΔCRP levels after delivery.*

| | | *Time after delivery (h)* | | | | | |
|---|---|---|---|---|---|---|---|
| | *Newborns* | *0* | *6* | *12* | *24* | *48* | *72* |
| CRP (ng/ml) | Non-infected ($n = 10$) | 59.1 ± 16.4 | 104.5 ± 28.6 | 405.3 ± 81.5 | 1268.1 ± 492.8 | 1117.5 ± 359.0 | 956.6 ± 150.0 |
| | Infected ($n = 26$) | 53.8 ± 10.1 | 265.7 ± 151.3 | 6713.0 ± 2987.0 | 8331.2 ± 2334.8** | 10504 ± 2907.5** | 4417.8 ± 1008.8** |
| ΔCRP (ng/ml/h) | Non-infected ($n = 10$) | | 4.7 ± 5.3 | 34.5 ± 9.6 | 50.8 ± 26.1 | 29.8 ± 8.5 | 15.1 ± 2.9 |
| | Infected ($n = 26$) | | 58.2 ± 35.9 | 548.9 ± 230.5* | 395.9 ± 114.2** | 306.5 ± 94.7** | 68.7 ± 16.1** |

Each value shows mean ± 1SE, * $P < 0.05$, ** $P < 0.001$.

CRP monitoring is very helpful for diagnosing infection, because large increases are sufficiently more likely to occur in cases of newborn infection than in healthy infants. Serial observation of CRP was both sensitive and reproductible, and detection of subtle changes with a minimum amount of blood was possible. When using CRP concentration as a parameter for diagnosis, a significant difference was observed between infected and non-infected groups at 24 h after delivery, and no subject in the non-infected group showed a CRP of 3000 ng/ml or higher. The $\Delta$CRP (ng/ml/h) could be a better parameter, since a significant difference was found at 12 h. Also, a threshold of about 70 $\Delta$CRP (ng/ml/h) could differentiate an infected from a non-infected newborn, with a sensitivity [true positive/(true positive + false negative)] of 69.9% and specificity [true negative/(true negative + false positive)] of 100%. Thus, this fully automated, non-isotopic method should be clinically useful for early detection of infection in newborns.

### References

1. Manroe, B. L., 1979, *J. Pediatr.*, **95**, 89.

2. Buck, C., Bundschu, J., Gallati, H., Bartmann, P. and Pohlandt, F., 1994, *Pediatr.*, **93**, 54.

3. Miller, L. C., Isa, S., Lopreste, G., Schaller, J. G and Dinarello, C. A., 1990, *J. Pediatr.*, **117**, 961.

4. Philip, A. G., 1984, *J. Pediatr.*, **105**, 940.

5. Mathers, N. J. and Pohlandt, F., 1987, *Eur. J. Pediatr.*, **146**, 147.

6. Sabel, K. G and Wadsworth, C. H., 1979, *Acta Paediatr. Scand.*, **68**, 825.

7. Shine, B., Gould, J., Campbell, C., Hindocha, P., Wilmot, P. and Wood, C. B., 1985, *Clin. Chim. Acta*, **148**, 97.

8. Hindocha, P., Campbell, C. A., Gould, J. D., Wojciechowski, A. and Wood, C. B., 1984, *Jpn J. Clin. Pathol.*, **37**, 1014.

9. Nishida, A., Ohtani, H., Saito, M., Ohmori, N. and Nishida, H., 1986, *J. Jpn Pediatr. Soc.*, **90**, 2691.

10. Yamagishi, Y., Sakurabayashi, I., Iwata, H. and Kawai, T., 1984, *Jpn J. Clin. Pathol.*, **32**, 1389.

11. Claus, D. R., Osmond, A. P. and Gewurz, H., 1976, *J. Lab. Clin. Med.*, **87**, 120.

12. Shine, B., Debeer, F. C. and Pepys, M. B., 1981, *Clin. Chim. Acta*, **117**, 13.

13. Niederau, C. H. M. and Reinauer, H., 1994, *Eur. J. Lab. Med.*, **2**, 117.

